# Coordinated Metabolic Transitions During *Drosophila* Embryogenesis and the Onset of Aerobic Glycolysis

**DOI:** 10.1534/g3.114.010652

**Published:** 2014-03-12

**Authors:** Jason M. Tennessen, Nicolas M. Bertagnolli, Janelle Evans, Matt H. Sieber, James Cox, Carl S. Thummel

**Affiliations:** * Department of Human Genetics, University of Utah School of Medicine, Salt Lake City, Utah 84112; † Scientific Computing and Imaging (SCI) Institute, University of Utah, Salt Lake City, Utah 84112; ‡ Department of Mathematics, University of Utah, Salt Lake City, Utah 84112; § Department of Biochemistry and the Metabolomics Core Research Facility, University of Utah, Salt Lake City, Utah 84112

**Keywords:** metabolism, aerobic glycolysis, embryogenesis, metabolomics

## Abstract

Rapidly proliferating cells such as cancer cells and embryonic stem cells rely on a specialized metabolic program known as aerobic glycolysis, which supports biomass production from carbohydrates. The fruit fly *Drosophila melanogaster* also utilizes aerobic glycolysis to support the rapid growth that occurs during larval development. Here we use singular value decomposition analysis of modENCODE RNA-seq data combined with GC-MS-based metabolomic analysis to analyze the changes in gene expression and metabolism that occur during *Drosophila* embryogenesis, spanning the onset of aerobic glycolysis. Unexpectedly, we find that the most common pattern of co-expressed genes in embryos includes the global switch to glycolytic gene expression that occurs midway through embryogenesis. In contrast to the canonical aerobic glycolytic pathway, however, which is accompanied by reduced mitochondrial oxidative metabolism, the expression of genes involved in the tricarboxylic cycle (TCA cycle) and the electron transport chain are also upregulated at this time. Mitochondrial activity, however, appears to be attenuated, as embryos exhibit a block in the TCA cycle that results in elevated levels of citrate, isocitrate, and α-ketoglutarate. We also find that genes involved in lipid breakdown and β-oxidation are upregulated prior to the transcriptional initiation of glycolysis, but are downregulated before the onset of larval development, revealing coordinated use of lipids and carbohydrates during development. These observations demonstrate the efficient use of nutrient stores to support embryonic development, define sequential metabolic transitions during this stage, and demonstrate striking similarities between the metabolic state of late-stage fly embryos and tumor cells.

The metabolism of proliferating cells must not only generate the energy that maintains cellular physiology but also provide precursors to synthesize the lipids, amino acids, and nucleotides required for rapid growth. This is particularly apparent in cancer cells, which rely on a metabolic program known as the Warburg effect or aerobic glycolysis to generate biomass (Warburg 1956; Vander Heiden *et al.* 2009). Aerobic glycolysis is characterized by the increased activity of glucose transporters, glycolytic enzymes, the pentose phosphate pathway, and other proteins that promote glycolytic flux. The resulting upregulation of glycolysis, however, is not solely used to produce ATP. Instead, the abundant supplies of glucose-derived metabolites are used to generate the amino acids, nucleotides, and fatty acids needed for biomass accumulation. Meanwhile, a significant quantity of the pyruvate generated during this process is not oxidized in the mitochondria, but rather is converted into lactate. This hallmark of aerobic glycolysis allows cells to regenerate the electron acceptor NAD^+^, which is required for maximal glycolytic flux. Interestingly, aerobic glycolysis is not restricted to cancer cells, but appears to be more widely used by proliferating cells, including human embryonic stem cells, lymphoblasts, and yeast grown under ideal culture conditions (Diaz-Ruiz *et al.* 2011; Michalek *et al.* 2011; Zhou *et al.* 2012).

The manner in which cancer cells rely on aerobic glycolysis suggests that exploring the molecular mechanisms that regulate this metabolic program could lead to new clinical therapies. While investigations using cancer cell lines are important for this endeavor, recent studies have demonstrated that tumor metabolism *in vivo* can differ significantly from these *in vitro* systems. For example, stable-isotope tracer experiments in transplanted glioblastoma multiforme (GBM) tumors reveal that glycolytic flux is linked with oxidative phosphorylation (Marin-Valencia *et al.* 2012). Consistent with this finding, oxidative phosphorylation is critical for maintaining GBM cancer stem cells (Janiszewska *et al.* 2012), and a recent survey of gene expression in GBM tumors revealed that genes encoding components of complex I in the electron transport chain (ETC) are among the most highly expressed transcripts in this tumor type (Bertagnolli *et al.* 2013). These discrepancies highlight the importance of studying aerobic glycolysis in intact animal models, in which growth and cell proliferation occur in the context of normal physiology.

We have shown previously that *Drosophila* larvae utilize aerobic glycolysis to support the remarkable growth that occurs during this stage, demonstrating that this metabolic state can be used in a developmental context and suggesting that *Drosophila* genetics can be exploited for understanding its regulation (Tennessen *et al.* 2011). The onset of aerobic glycolysis in *Drosophila* occurs approximately 12 hr before the end of embryogenesis, when the *Drosophila* Estrogen-Related Receptor (dERR) triggers the coordinate transcriptional upregulation of nearly every gene that encodes an enzyme involved in glycolysis, as well as *Lactate Dehydrogenase* (*Ldh*, also known as *ImpL3* in *Drosophila*) (Tennessen *et al.* 2011). This embryonic metabolic transition (EmbMT) allows newly hatched larvae to efficiently convert dietary carbohydrates into biomass, thereby supporting the nearly 200-fold increase in body mass that occurs during the 4 d of larval development. When aerobic glycolysis is inhibited during this growth phase, such as in *dERR* or *Phosphofructokinase* mutants, larvae are unable to metabolize sufficient quantities of sugar and die during the second larval instar (Tennessen *et al.* 2011).

The transcriptional induction of aerobic glycolysis occurs at a highly reproducible time point during *Drosophila* embryogenesis, providing an opportunity to understand its regulation and function in the context of normal physiology. As a first step toward defining the metabolic changes that accompany the onset of this metabolic program, we have coupled two powerful approaches to systematically analyze the metabolic state of *Drosophila* embryogenesis. The modENCODE project has previously reported the comprehensive transcriptional profiling of staged *Drosophila* embryos using RNA-seq (Graveley *et al.* 2011). We have analyzed these data using singular value decomposition (SVD) to identify significant patterns of gene expression. This mathematical technique is ideally suited for identifying patterns in large datasets (Alter 2006) and has been used successfully to identify co-expressed genes in microarray studies, to correlate gene expression with cell cycle progression, and to determine how mRNA transcript length is correlated with tumor metabolism (Alter *et al.* 2000; Alter and Golub 2004; Alter and Golub 2006; Li and Klevecz 2006; Bertagnolli *et al.* 2013). Here we use SVD to identify the transcriptional programs that are coordinately regulated during embryogenesis and uncover a correlative relationship between the upregulation of genes that encode glycolytic enzymes and components of the tricarboxylic acid (TCA) cycle and ETC. This analysis of gene expression was complemented by a comprehensive metabolomic analysis of staged *Drosophila* embryos. Our GC-MS-based analysis of approximately 100 polar metabolites identified the unexpected accumulation of key molecules associated with glycolysis, the TCA cycle, and nucleotide degradation, as well as the depletion of aspartate and kynurenine. These observations suggest that the TCA cycle is partially repressed and that embryos are pre-adapted to oxidative stress. In addition, our study reveals clear parallels between the onset of aerobic glycolysis in *Drosophila* embryos and metabolic factors that are known to promote tumor growth.

## Materials and Methods

### *Drosophila* strain selection

All studies were conducted using Canton-S (*CanS*) and *w^1118^* strains that have been maintained in the Thummel laboratory and extensively used for metabolic studies (Sieber and Thummel 2009; Wang *et al.* 2010; Tennessen *et al.* 2011; Bricker *et al.* 2012). The modENCODE RNAseq data used for the gene expression analysis was generated from an isogenic *y^1^*; *cn bw^1^sp^1^* strain (Brizuela *et al.* 1994; Graveley *et al.* 2011). This strain, however, is not appropriate for metabolomic analysis because *cn* encodes a kynurenine 3-monooxygenase that is involved in tryptophan metabolism (Sullivan *et al.* 1973), *y* mutants exhibit defects in lysine and tryptophan metabolism (Bratty *et al.* 2012), and *sp* is a known regulator of phenol oxidase (Warner *et al.* 1975).

### Statistical analysis of embryonic gene expression

The comprehensive RNA-seq data from staged embryos reported in the Supplemental Table 17 of Graveley *et al.* (2011) were analyzed by SVD as described previously (Alter *et al.* 2000; Bertagnolli *et al.* 2013). All calculations were conducted using the program Mathematica 9 (Wolfam) (Supporting Information, File S1). This mathematical technique transforms the data from (transcript) × (time space) to (eigengenes) × (eigentime), with the new space represented by the matrices:

$$D=USV^{T}$$

To identify the most significant eigengenes, the eigenexpression $e_{i}$ was calculated by dividing each of the singular values in *S* by the sum of the singular values:

$$e_{i}=\frac{S_{ii}}{\sum_{i=1}^{12}S_{ii}}$$

These new fractional abundances were then used to find the Shannon entropy and assess the significance of the i^th^ eigengene, where a 0 represents highly ordered data and 1 represents highly unordered data.

$$0\leq d=\frac{1}{-\text{log}(12)}\sum_{i=1}^{12}e_{i}\text{log}(e_{i})\leq 1$$

To determine which genes were most closely associated with each eigengene observed in the *V^T^* matrix, columns of the *U* matrix were sorted in descending order and the top 500 genes were analyzed for gene ontology enrichment using GOrilla (Eden *et al.* 2007; Eden *et al.* 2009). Hypergeometric distribution was used to determine if there was a significant enrichment for metabolic genes among the top 500 genes associated with each eigengene pattern (Tavazoie *et al.* 1999). This statistical method determines the probability of having k success in m draws where there are K possibilities of success in some set of size M, and was calculated using the following equation:

$$\sum_{i=k}^{m}\frac{\left(\begin{array}{c} K\\ i \end{array}\right)\left(\begin{array}{c} M-K\\ m-i \end{array}\right)}{\left(\begin{array}{c} M\\ m \end{array}\right)}$$

For our analysis, there are 17,000 genes in the modENCODE dataset (M) and 1228 metabolic genes (K; Table S2); k is the number of genes selected with a metabolic annotation present within the top 500 genes (m) associated with each SVD pattern.

### Northern blots

Staged *w^1118^* embryos were collected, staged, and dechorionated, and RNA was extracted using Trizol Reagent (LifeTechnologies). Samples of 3 µg total RNA were used for Northern blot analysis, essentially as described (Karim and Thummel 1991).

### Glycogen and triglyceride assays

Staged embryos were collected at 6-hr intervals from population cages of either *w^1118^* or *CanS* flies. Six samples containing 300 embryos were collected for each time point. For triglyceride assays, embryos were placed in a screw-cap tube containing 200 µl of PBS, 0.05% Tween 20 with 1.4-mm ceramic beads (BioExpress), and flash-frozen in liquid nitrogen. Samples were homogenized using an Omni Bead Ruptor (Omni International) and heat-treated at 70° for 5 min. A 20-µl sample of the homogenate was used to measure triglyceride concentration as described previously (Palanker *et al.* 2009). For glycogen assays, 300 embryos were placed in a screw-cap tube containing 200 µl of PBS with 1.4-mm ceramic beads that were preheated to 70°. Samples were incubated at 70° for 5 min, flash-frozen in liquid nitrogen, and homogenized as described above. The homogenate was diluted 1:5 and the glycogen concentration was measured as described previously (Palanker *et al.* 2009).

### Embryo collections

For metabolomic analysis, 12 time points of *w^1118^* and *CanS* embryos, staged at 2-hr intervals throughout embryogenesis, were collected from seven sample sets derived from three independent matings, with each mating containing 200 females and 200 males. Embryos were harvested and staged on molasses-based egg caps containing a small amount of yeast paste. These data are included in Table S6 and Table S7. A technical difficulty with the gas chromatography-mass spectrometry (GC-MS) rendered three of the *CanS* sample sets unusable, and these data were omitted from our final analysis. An additional collection of staged *CanS* embryos was made from a population cage, with embryos harvested at 2-hr intervals from trays of semi-defined media (Backhaus *et al.* 1984) (Table S8). For all samples, 300 embryos were placed on a 55-mm disc of Whatmann 1 filter paper, washed with PBS, and then placed in a screw-cap tube containing 1.4-mm ceramic beads (BioExpress). Samples were frozen in liquid nitrogen and processed by the University of Utah metabolomics core facility as described below.

### Metabolite extraction and derivatization

Stable-isotope labeled internal standards, Cell Free Amino Acid Mix (Cambridge Isotope, catalog # CNLM-6696-1) and Succinic acid-2,2,3,3-d_4_ (Sigma, catalog # 293075), along with 500 µl of 90% cold methanol, were added to each sample. Samples were homogenized for 30 sec at 6.45 m/s in an Omni Bead Ruptor, centrifuged for 5 min at 20,000×*g*, and the supernatant was transferred to a 1.5-ml tube and dried. These samples were resuspended in 40 µl of 40 mg/ml O-methoxylamine hydrochloride in pyridine and incubated for 1 hr at 30°.

### GC-MS analysis

GC-MS analysis was performed with a Waters GCT Premier mass spectrometer fitted with an Agilent 6890 gas chromatograph and a Gerstel MPS2 autosampler. Twenty-five µl of derivatized solution was added to autosampler vials, followed by the addition of 40 μl of N-methyl-N-trimethylsilyltrifluoracetamide (MSTFA), after which the samples were incubated for 60 min at 37° with shaking. One µl of the sample was injected into the gas chromatograph inlet in the split mode at a 10:1 split ratio with the inlet temperature held at 250°. The gas chromatograph had an initial temperature of 95° for 1 min followed by a 40°/min ramp to 118° and a hold time of 2 min. This was followed by a second 5°/min ramp to 250°, a third ramp to 350°, and then a final hold time of 3 min. To increase the dynamic range of the detected metabolome and to recover data from detector saturated peaks, a second 100:1 injection was performed using the following ramp: an initial temperature of 95° for 1 min followed by a 40°/min ramp to 118° and a hold time of 1 min. This was followed by a second 25°/min ramp to 330°. A 30-m Phenomenex-ZB5MSi column with a 5-m guard column was used for chromatographic separation. Data were collected using MassLynx 4.1 software (Waters). Known metabolites were identified and their peak area was recorded using QuanLynx. Data were transferred to an Excel file where each sample was normalized to the internal standard D4-succinate. Statistical significance between the 0- to 2-hr sample and all subsequent samples were determined using an unpaired T-test with the Welch correction.

## Results

### The transcriptional initiation of aerobic glycolysis is coordinated with mitochondrial metabolism and fatty acid breakdown

**Figure 1:**
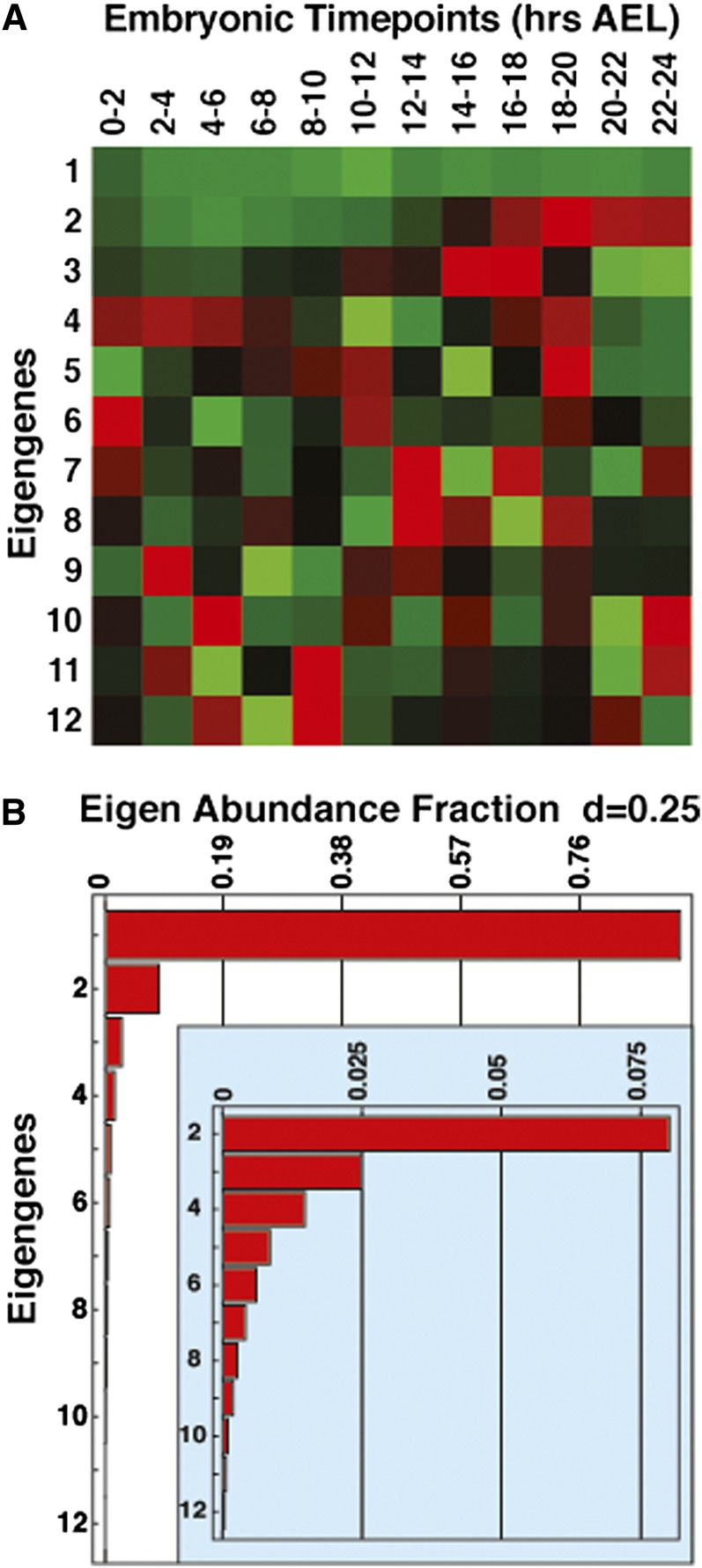
Statistical analysis of embryonic gene expression using SVD. Embryonic RNA-seq time course data from the *Drosophila* modENCODE project were analyzed using SVD, revealing (A) 12 eigengene expression patterns in matrix *V^T^*. (B) A bar graph depicts the eigenexpression fractions demonstrating that the top three patterns account for 96% of the overall expression in embryos. Consistent with this observation, the data possess a low Shannon entropy (d = 0.25), indicating that the majority of the data are characterized by a subset of these patterns.

Our previous studies determined that nearly all genes involved in glycolysis, as well as *Ldh*, are induced midway through embryogenesis (Tennessen *et al.* 2011). To determine if other metabolic pathways are coordinately regulated during this stage, the large-scale gene expression dataset available from the *Drosophila* modENCODE project was analyzed using SVD (Table S1) (Graveley *et al.* 2011). This study revealed 12 eigengenes of coordinated gene expression among the 17,000 analyzed genes (Figure 1A). We further characterized the genes associated with the top three SVD patterns, which represent 96% of the overall expression in embryos (Figure 1B). Of these, the most statistically significant pattern represents genes that are expressed at a constant level throughout embryogenesis (Figure 1, A and B), a result that is expected for this type of analysis. In contrast, the next two most significant patterns of gene expression reveal coordinated changes in the expression of key genes involved in metabolism.

**Figure 2:**
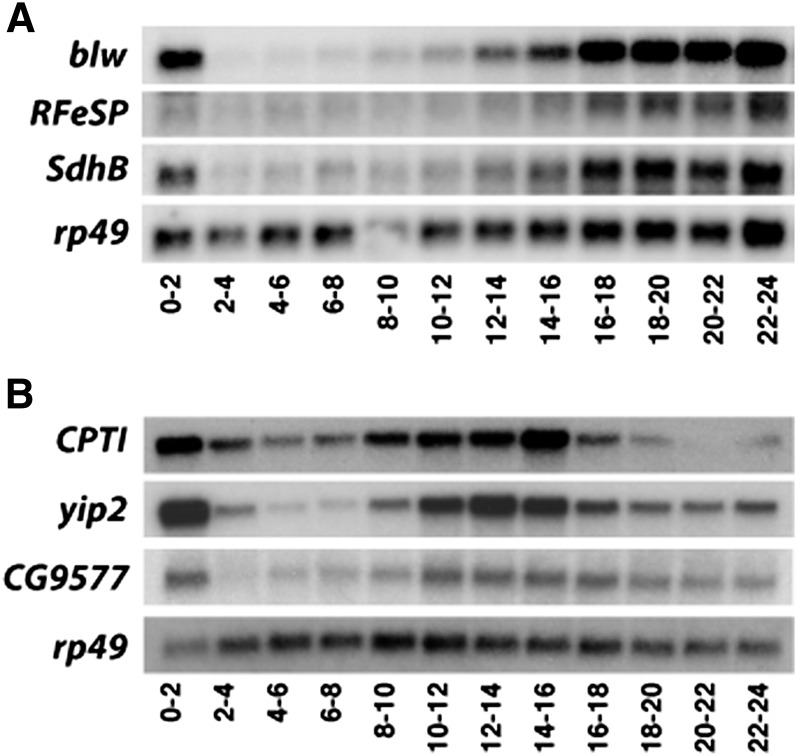
Transcriptional profiles of metabolic genes identified by SVD analysis. Total RNA from staged *w^1118^* embryos were analyzed by northern blot hybridization to detect transcripts encoding components of (A) the TCA cycle and ETC or (B) fatty acid β-oxidation. A transfer artifact makes the *CPTI* signal at 20 to 22 hr appear lower than it is; it should appear similar to the signal in the flanking lanes. Hybridization to detect *rp49* mRNA is included as a loading control.

The second most significant pattern identified by SVD corresponds to broad upregulation of gene expression midway through embryogenesis and is strikingly similar to the coordinate induction of glycolytic genes that defines the EmbMT (eigengene pattern 2, Figure 1A). Consistent with this, Gene Ontology (GO) analysis of the top 500 genes associated with this pattern revealed that the most significant category corresponds to the Generation of Precursor Metabolites and Energy (GO:0006091) (Table 1). In addition, these 500 genes include every gene that encodes a glycolytic enzyme, *Ldh*, and *Pepck* (Table 2). A number of genes involved in mitochondrial metabolism are also present on this list, including genes that encode a pyruvate dehydrogenase complex subunit, ETC subunits, and enzymes in the TCA cycle. A more focused functional analysis of these 500 genes was achieved by comparing them with a list of 1228 genes that either are known metabolic regulators or are listed in the KEGG metabolic pathways for *Drosophila* (Table S2). This analysis revealed that metabolic genes are significantly enriched in pattern 2 (*P* = 1.10×10^−20^; hypergeometric distribution), with 99 of the top 500 genes associated with cellular metabolism, including a variety of ETC and TCA cycle components that were not included in the Generation of Precursor Metabolites and Energy GO category (Figure S1A, Table S3). Several of these EmbMT-associated genes were selected for validation by northern blot hybridization using RNA samples from staged *CanS* and *w^1118^* embryos (Figure 2A), including the ETC complex V member encoded by *blw*, the predicted ubiquinol-cytochrome c reductase encoded by *R*F*eSP*, and the *SdhB*-encoded subunit of the succinate dehydrogenase complex that acts in the TCA cycle. All three of these genes display temporal expression patterns that reflect this SVD category and that parallel the global induction of glycolytic gene expression that defines the EmbMT (Figure 2A).

**Table 1:** Top 25 GO categories present within SVD eigengene pattern 2

**GO Term**	**Description**	**B***^a^*	**b***^b^*	***P***
GO:0006091	Generation of precursor metabolites and energy	114	37	4.95E−27
GO:0006818	Hydrogen transport	57	26	8.12E−24
GO:0015992	Proton transport	57	26	8.12E−24
GO:0022900	Electron transport chain	72	24	3.21E−18
GO:0022904	Respiratory electron transport chain	68	23	1.13E−17
GO:0015988	Energy coupled proton transmembrane transport	35	17	1.44E−16
GO:0015991	ATP hydrolysis coupled proton transport	35	17	1.44E−16
GO:0046034	ATP metabolic process	29	15	2.71E−15
GO:0034220	Ion transmembrane transport	117	25	6.79E−14
GO:0015672	Monovalent inorganic cation transport	147	27	3.26E−13
GO:0055114	Oxidation-reduction process	485	50	7.01E−13
GO:0006754	ATP biosynthetic process	22	12	7.64E−13
GO:0044710	Single-organism metabolic process	1246	89	1.20E−12
GO:0015985	Energy coupled proton transport	18	11	1.27E−12
GO:0015986	ATP synthesis coupled proton transport	18	11	1.27E−12
GO:0006006	Glucose metabolic process	43	15	3.46E−12
GO:1901135	Carbohydrate derivative metabolic process	344	40	3.98E−12
GO:0010171	Body morphogenesis	20	11	6.32E−12
GO:0009126	Purine nucleoside monophosphate metabolic process	45	15	7.41E−12
GO:0009167	Purine ribonucleoside monophosphate metabolic process	45	15	7.41E−12
GO:0009206	Purine ribonucleoside triphosphate biosynthetic process	26	12	1.01E−11
GO:0009145	Purine nucleoside triphosphate biosynthetic process	26	12	1.01E−11
GO:0009201	Ribonucleoside triphosphate biosynthetic process	27	12	1.77E−11
GO:0009142	Nucleoside triphosphate biosynthetic process	27	12	1.77E−11
GO:0009123	Nucleoside monophosphate metabolic process	51	15	5.70E−11

a B refers to the number of genes associated with a GO term.

b b refers to the number of genes in the target list that are also associated with a specific GO term.

**Table 2:** Genes present in SVD pattern 2 associated with GO category GO:0006091 Generation of Precursor Metabolites and Energy

**CG**	**Gene**	**Function**	**Metabolic Pathway**
**CG17246**	***SdhA***	Succinate dehydrogenase	Citric acid cycle
**CG3283**	***SdhB***	Succinate dehydrogenase	Citric acid cycle
**CG6666**	***SdhC***	Succinate dehydrogenase	Citric acid cycle
**CG14482**	***CG14482***	Ubiquinol-cytochrome c reductase subunit 10	Electron transport
**CG3560**	***CG3560***	Ubiquinol-cytochrome c reductase subunit 7	Electron transport
**CG4169**	***CG4169***	Ubiquinol-cytochrome c reductase core subunit 2	Electron transport
**CG4769**	***CG4769***	Ubiquinol-cytochrome c reductase cytochrome c1 subunit	Electron transport
**CG6020**	***CG6020***	NADH dehydrogenase (ubiquinone) 1 alpha subcomplex subunit 9	Electron transport
**CG7580**	***CG7580***	Cytochrome b-c1 complex subunit 8	Electron transport
**CG9140**	***CG9140***	NADH dehydrogenase	Electron transport
**CG14724**	***CoVa***	Cytochrome c oxidase subunit Va	Electron transport
**CG2249**	***CoVIIc***	Cytochrome c oxidase subunit VIIc	Electron transport
**CG7181**	***CoVIII***	Cytochrome c oxidase subunit VIII	Electron transport
**CG14028**	***cype***	Cyclope; Cytochrome c oxidase subunit 6C	Electron transport
**CG17903**	***Cyt-c-p***	Cytochrome c proximal	Electron transport
**CG17280**	***levy***	Levy; Cytochrome c oxidase, subunit Via	Electron transport
**CG9160**	***mtacp1***	Mitochondrial acyl carrier protein 1	Electron transport
**CG2286**	***ND75***	NADH:ubiquinone reductase 75kD subunit precursor	Electron transport
**CG8764**	***ox***	Oxen; Cytochrome b-c1 complex subunit 9	Electron transport
**CG7361**	***RFeSP***	Rieske iron-sulfur protein	Electron transport
**CG5320**	***Gdh***	Glutamate dehydrogenase	Glutamate metabolism
**CG7254**	***GlyP***	Glycogen phosphorylase	Glycogenolysis
**CG17654**	***Eno***	Enolase	Glycolysis
**CG12055**	***Gapdh1***	Glyceraldehyde phosphate dehydrogenase	Glycolysis
**CG8893**	***Gapdh2***	Glyceraldehyde phosphate dehydrogenase	Glycolysis
**CG8251**	***Pgi***	Phosphoglucose isomerase	Glycolysis
**CG3127**	***Pgk***	Phosphoglycerate kinase	Glycolysis
**CG1721**	***Pglym78***	Phosphoglycerate mutase	Glycolysis
**CG7070**	***Pyk***	Pyruvate kinase	Glycolysis
**CG2171**	***Tpi***	Triose phosphate isomerase	Glycolysis
**CG10160**	***ImpL3***	Lactate dehydrogenase	Lactate synthesis
**CG7010**	***l(1)G0334***	Pyruvate dehydrogenase E1 component	Pyruvate metabolism

Pattern three identified by SVD represents an expression profile that decreases in mid embryogenesis and increases in late embryogenesis (Figure 1A). Although this pattern is not enriched for metabolic genes (*P* = 0.17 for top 500 genes; Figure S1B), there is a significant enrichment for genes associated with chitin formation (GO:0042335, GO:0040003), which is consistent with the timing of embryonic cuticle deposition (Table S4). Moreover, a closer examination of the top 500 genes associated with pattern 3 uncovered genes involved in lipid metabolism, including the rate-limiting enzyme for β-oxidation, CPTI, and the Lip1 lipase (Figure 2B, Table S5). The expression of *CPTI* was validated by northern blot hybridization, revealing upregulation in 8- to 10-hr embryos and repression at 16 to 18 hr (Figure 2B). A similar temporal pattern of expression was seen when other β-oxidation genes were examined by northern blot analysis, including *yip2* and *CG9577* (Figure 2B). These observations suggest that fatty acid oxidation plays an important role in energy production during mid embryogenesis and supports previous observations that insect embryos use lipid metabolism to drive developmental progression (Medina and Vallejo 1989). It is interesting to note that these genes are coordinately downregulated in late embryos in parallel with the onset of the EmbMT, suggesting that the embryo is switching its metabolic gene expression program from fatty acid breakdown to aerobic glycolysis in preparation for hatching.

### Embryonic depletion of maternal energy stores

**Figure 3:**
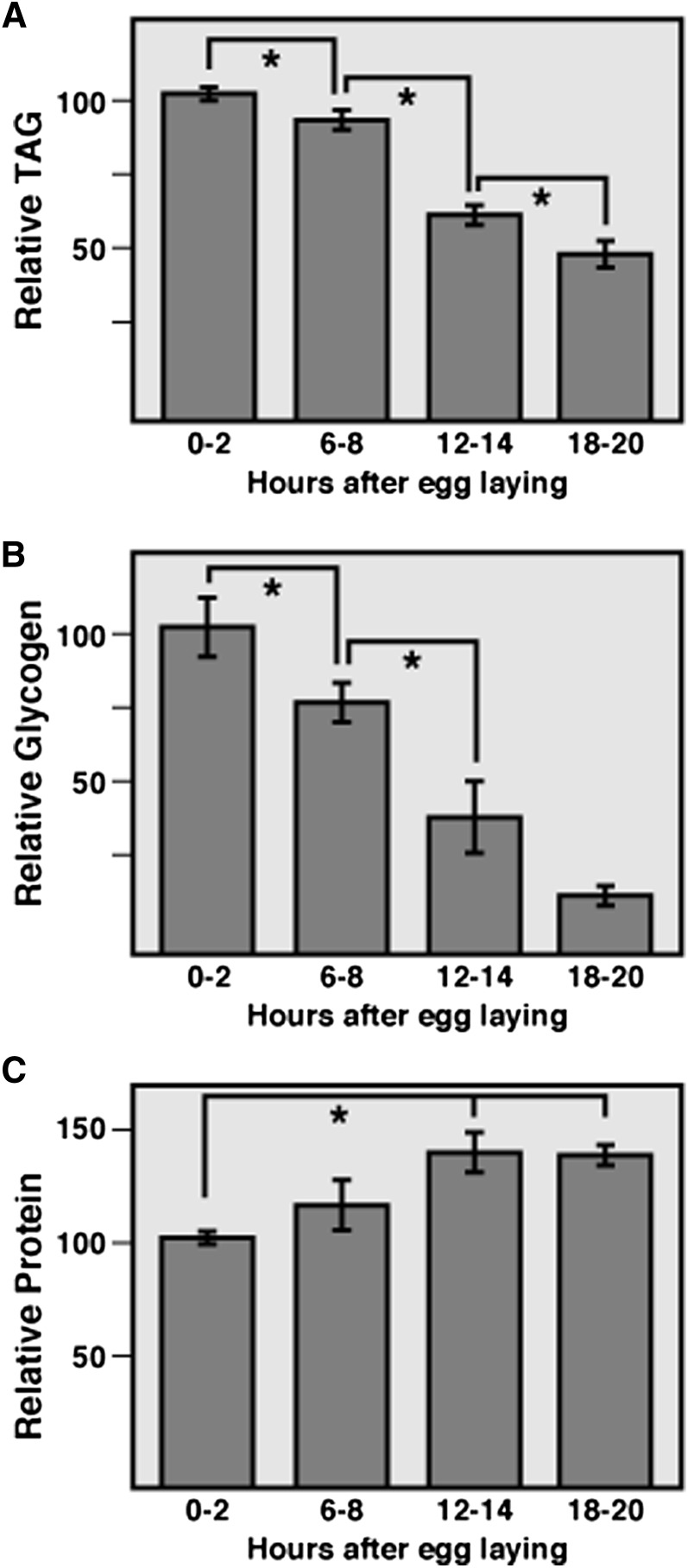
Stored lipids and carbohydrates are depleted during *CanS* embryogenesis. Triacylglycerol (TAG) (A), glycogen (B), and soluble protein (C) levels were measured at 4-hr intervals during the course of embryogenesis. Both glycogen and TAG exhibit a significant decrease as development progresses, whereas soluble protein levels increase. Each bar represents the mean value of n = 6 samples containing 300 staged and hand-sorted *CanS* embryos. Data were normalized to the mean value of the 0- to 2-hr time point. *P* < 0.05, Student t-test). Error bars represent ± SEM.

The coordinated changes in the expression of genes involved in glycolysis, β-oxidation, and mitochondrial metabolism suggest that both carbohydrates and lipids are being used as energy sources to sustain embryonic development. As a means of testing this hypothesis, we examined the levels of maternally deposited stores of glycogen and triglyceride (TAG) at four 2-hr time points spanning embryogenesis. Both *CanS* (Figure 3) and *w^1118^* strains (Figure S2) exhibited a steady decrease in both energy pools, consistent with the proposal that embryos are utilizing these reserves of fat and carbohydrates to support development (Medina and Vallejo 1989; Vital *et al.* 2010). In contrast, soluble protein levels exhibit a modest increase during *CanS* development (Figure 3C).

### Metabolomic profiling of *Drosophila* embryogenesis

The breakdown of glycogen and TAG suggest that developing embryos are using these reserves to derive the energy required to complete embryonic development as well as to generate the biomolecules needed for cellular differentiation. To further explore how embryos utilize maternally deposited nutrients and determine how metabolic homeostasis changes at the onset of aerobic glycolysis, we conducted a metabolomic survey of embryogenesis using a GC-MS-based approach. Our analysis examined the relative concentration of more than 100 polar compounds at 2-hr intervals throughout the course of embryonic development (Table S6, Table S7, Table S8). Here we focus on key metabolites associated with central metabolism, as well as those compounds that change consistently across three independent time course experiments.

### Temporal profiling of glycolytic metabolites

**Figure 4:**
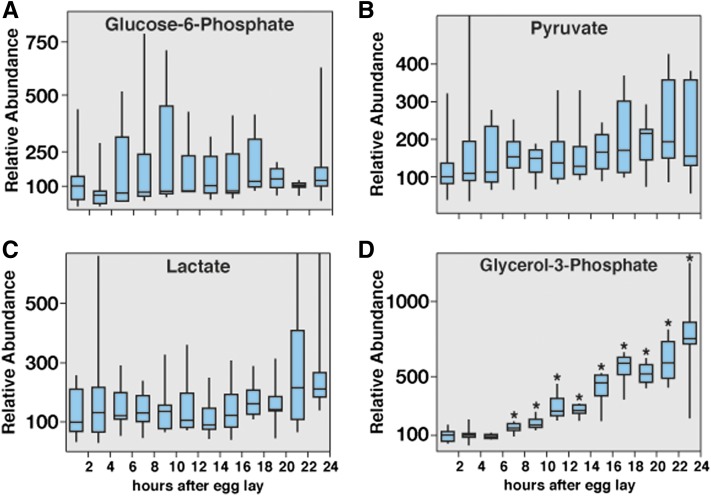
Metabolomic analysis of glycolysis in *w^1118^* embryos. Small-molecule GC-MS was used to analyze the relative abundance of metabolites related to glycolysis. (A) Although glucose-6-phosphate concentrations exhibited significant fluctuation, the median value remained nearly constant throughout embryogenesis. (B, C) Both pyruvate and lactate levels increase gradually as embryogenesis progresses, although these changes are not significant. (D) Embryos exhibit a nearly 10-fold increase in glycerol-3-phosphate levels. All data are graphically represented as a box plot, with the box representing the first and third quartiles, the median represented as the horizontal line within the box, and the bars representing the maximum and minimum points. Values are relative to the median of the 0- to 2-hr sample, which was normalized to 100; n > 6 independent samples for each time point. Each sample contains 300 staged and hand-sorted embryos. *P < 0.01 compared with the 0- to 2-hr AEL time point.

We were able to detect a number of metabolites associated with glycolysis, including glucose-6-phosphate, pyruvate, and lactate (Figure 4). In our first two sets of samples, the onset of the EmbMT did not significantly alter the levels of these metabolites (Figure 4, A–C, Table S6, Table S7). Whereas glucose-6-phosphate levels exhibited large variations during our analysis, the median value remained relatively stable. Furthermore, although both lactate and pyruvate levels appear to increase during the course of embryogenesis, the lactate-to-pyruvate ratio remains constant. The third set of samples, however, exhibited more significant changes in these metabolites, including an approximately six-fold increase in the concentration of glucose-6-phosphate and lactate (Figure S3, A–C and Table S8). This apparent discrepancy is likely a function of parental diet, because the first two time course experiments were collected from parents that were maintained on yeast paste as a food source, whereas the third set of samples was collected from parents maintained on semi-defined medium, which contains a higher sugar content (Backhaus *et al.* 1984).

The glycolytic metabolite that changes most significantly during embryogenesis is glycerol-3-phosphate (G3P) (Figure 4D, Figure S3D), which can be interconverted with the glycolytic intermediate dihydroxyacetone phosphate (DHAP). We observed that G3P levels start to increase 6 to 8 hr after egg laying (AEL) and then undergo a dramatic five-fold to 10-fold increase during the remainder of embryogenesis.

### Temporal profiling of TCA cycle intermediates

**Figure 5:**
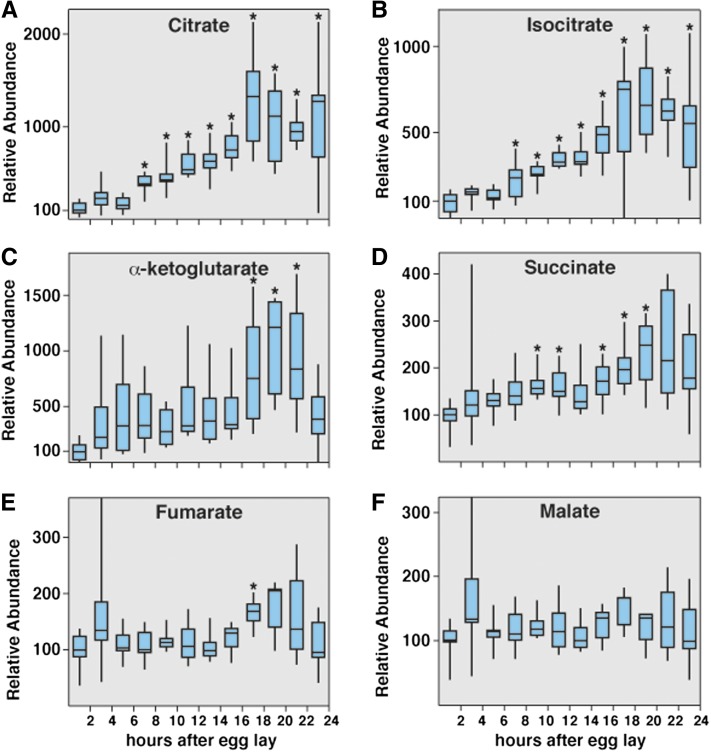
Metabolomic analysis of TCA cycle intermediates in *w^1118^* embryos. Small-molecule GC-MS was used to analyze the relative abundance of TCA cycle intermediates. (A–C) Citrate, isocitrate, and α-ketoglutarate levels significantly increase during the course of embryogenesis. In contrast, the concentration of succinate (D) approximately doubles during this time course, whereas fumarate (E) and malate (F) levels remain relatively stable. All data are graphically represented as described in Figure 4. *P < 0.01 compared with the 0- to 2-hr AEL time point.

Our metabolomic study detected nearly every TCA cycle intermediate, with the exception of oxaloacetate and CoASH derivatives. Although SVD analysis revealed that many of the genes that encode the TCA cycle enzymes gradually increase in expression during the course of embryogenesis, the abundance of TCA cycle intermediates in staged embryos suggest that there is a block in this metabolic pathway. The concentration of citrate undergoes a greater than five-fold increase during embryogenesis (Figure 5A, Table S6, Table S7, Table S8). Similarly, isocitrate and α-ketoglutarate are nearly undetectable at the onset of embryogenesis, but increase dramatically during the course of embryogenesis (Figure 5, B and C and Table S6, Table S7, Table S8). In contrast, succinate exhibits a modest increase, whereas both fumarate and malate levels remain largely constant during the course of our analysis (Figure 5, D–F and Table S6, Table S7, Table S8). Taken together, these results suggest that there is reduced flux through the TCA cycle during late embryogenesis as the aerobic glycolytic program is being induced.

### Temporal profiling of amino acids

**Figure 6:**
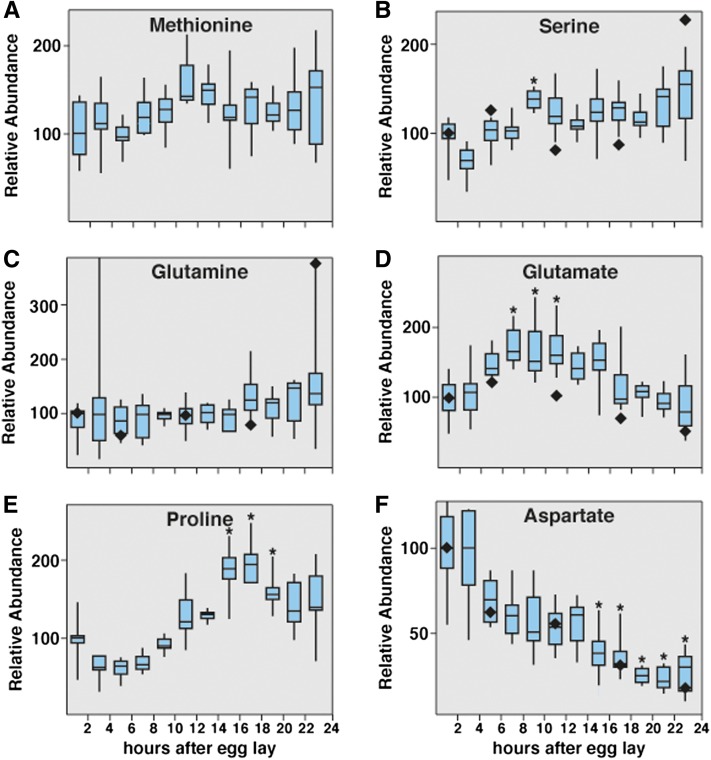
Changes in *w^1118^* embryonic amino acid pools. Small-molecule GC-MS was used to analyze changes in amino acid levels at 2-hr intervals throughout the course of *w^1118^* embryogenesis. The essential amino acid methionine (A) as well as glucogenic amino acid serine (B) and the ketogenic amino acid glutamine (C) exhibit only minor fluctuations during the course of embryogenesis. The abundance of glutamate (D) increases during the beginning of embryogenesis, but then gradually declines until just prior to hatching. In contrast, proline (E) decreases during early embryogenesis and then increases approximately two-fold compared with the initial concentration. (F) Aspartate undergoes a consistent and dramatic decrease throughout the course of embryogenesis. All data are graphically represented as described in Figure 4. *P < 0.01 compared with the 0- to 2-hr AEL time point. Black diamonds (♦) represent the relative amino acid concentrations reported by Crone-Gloor (1959).

**Figure 7:**
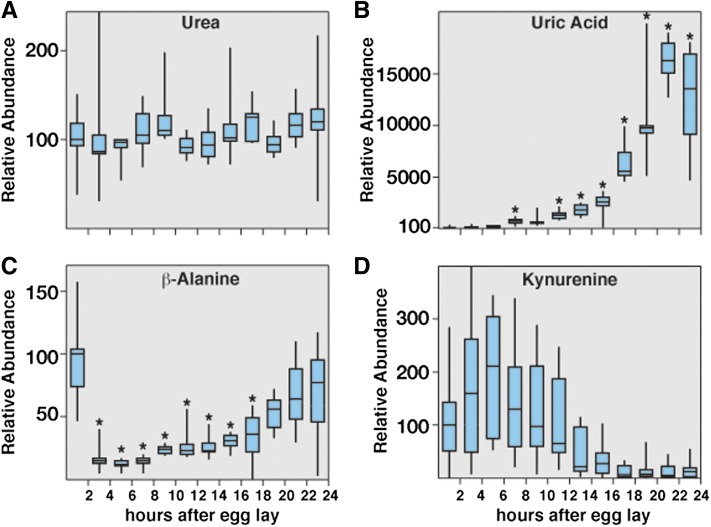
Analysis of metabolites associated with amino acid and purine degradation in *w^1118^* embryos. Small-molecule GC-MS was used to analyze the relative abundance of compounds associated with amino acid and purine degradation at 2-hr intervals throughout the course of *w^1118^* embryogenesis. Although the levels of urea (A) remain stable throughout embryogenesis, uric acid levels (B) exhibit the most dramatic increase of any metabolite in our analysis. (C) β-alanine levels decline sharply 2 to 4 hr AEL and then gradually increase during the course of embryogenesis. (D) The relative concentration of kynurenine remains stable for the first 12 hr of embryogenesis but then undergoes a dramatic decrease that correlates with the onset of the EmbMT. All data are graphically represented as described in Figure 4. *P < 0.01 compared with the 0- to 2-hr AEL time point.

In general, the concentrations of essential amino acids such as methionine, valine, isoleucine, and leucine increase approximately 1.5-fold to 3-fold during embryogenesis (Figure 6A, Table S6, Table S7, Table S8). Because *Drosophila* is unable to synthesize these molecules, this increase must reflect a net degradation of maternal protein and suggests that protein turnover makes a modest contribution to the observed changes in amino acid concentration. Similarly, many nonessential amino acids, including serine, glutamine, and alanine, exhibit a similar overall increase during this 24-hr period (Figure 6, B and C, and Table S6, Table S7, Table S8), indicating that the synthesis and degradation of these amino acids remain at equilibrium throughout our analysis. There are three amino acid pools, however, that exhibit significant and reproducible changes during the course of embryogenesis. Glutamate levels increase during early embryogenesis and then gradually decline during the rest of development (Figure 6D). In contrast, proline abundance is inversely correlated with that of glutamate, undergoing a decline until 6 to 8 hr AEL, when it begins to increase (Figure 6E). Because glutamate can be interconverted with α-ketoglutarate or proline, both of which increase while glutamate levels decrease (Figure 5C, Figure 6E), the reciprocal changes in glutamate and proline may be related to the reduced metabolic flux through the TCA cycle at the end of embryogenesis. Finally, aspartate exhibits a nearly 10-fold decrease throughout the course of embryogenesis (Figure 6F), suggesting that this amino acid plays a central role in embryonic metabolism. Although the metabolic function of aspartate remains unclear in embryos, it is unlikely that this depletion is related to the urea cycle, as insects excrete nitrogenous waste in the form of uric acid, and urea concentrations remain unchanged throughout development (Figure 7A). These changes in embryonic amino acid levels are similar to measurements reported by Crone-Gloor (1959), providing external validation that our observations are accurate (Figure 6, B–D, F).

### Uric acid, β-alanine, and kynurenine levels change dramatically during embryogenesis

In addition to the amino acids and metabolites associated with central metabolism, we identified three compounds that undergo major fluctuations during embryogenesis. Uric acid levels increase nearly 100-fold during the second half of embryogenesis (Figure 7B), suggesting that late-stage embryos rely on amino acid and purine degradation for some energy production. However, none of the nucleotides and derivatives that we detected (nor overall protein) displayed a significant reduction, leaving the origin of the uric acid unclear. β-alanine levels undergo a rapid 10-fold decrease during the first 4 hr AEL, but then increase 10-fold over the remainder of embryogenesis (Figure 7C). Finally, embryos exhibit a 98% decrease in kynurenine at the onset of the EmbMT, which is the largest decrease of any metabolite in our analysis and suggests that degradation of this compound might be associated with the onset of aerobic glycolysis (Figure 7D).

## Discussion

Although *Drosophila* larval development relies on aerobic glycolysis to generate biomass, the transcriptional onset of this metabolic program occurs during embryonic development, nearly 12 hr prior to the beginning of growth. The timing of this metabolic switch, therefore, provides a unique opportunity to explore the metabolic changes that occur before, during, and after the onset of aerobic glycolysis. To exploit this system, we have conducted a survey of *Drosophila* embryonic metabolism that includes transcriptional profiling and metabolomic characterization of staged embryos. Our results not only provide a comprehensive description of embryonic metabolism but also reveal clear parallels between *Drosophila* development and tumor growth.

### Similarities between tumor metabolism and *Drosophila* development

**Figure 8:**
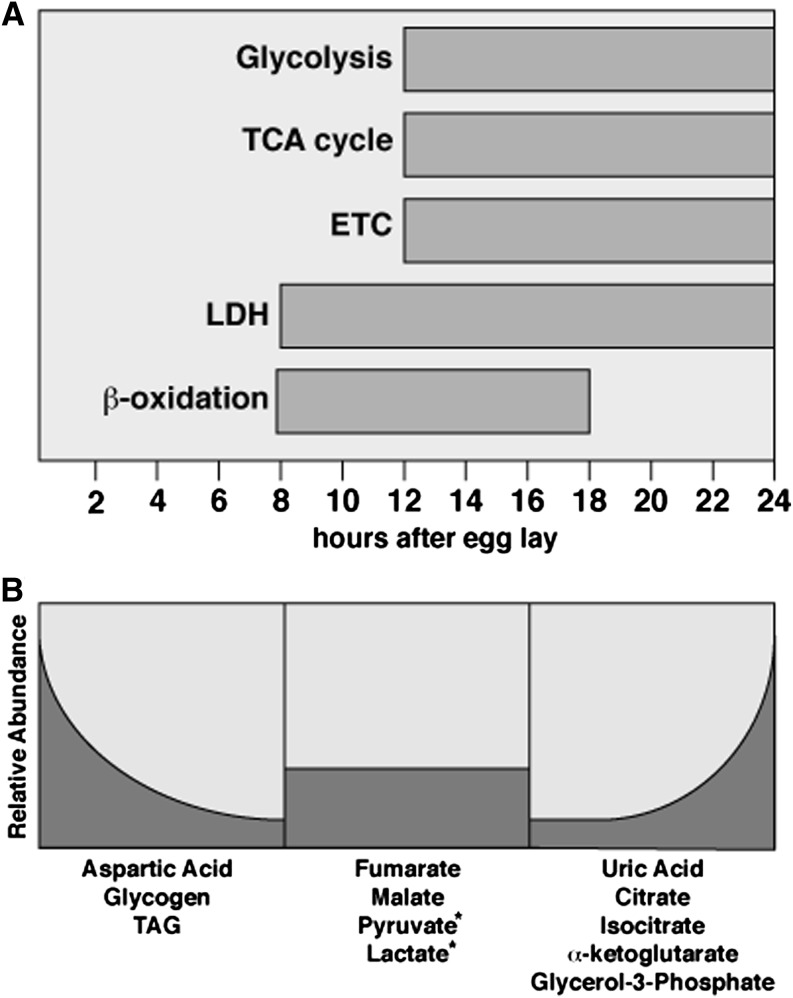
Summary of embryonic metabolism. (A) The coordinate upregulation of glycolysis during the EmbMT corresponds with the elevated expression of genes associated with the TCA cycle and ETC. (B) The expression of these genes correlate with the depletion of glycogen and the build-up of key metabolic intermediates, including citrate, isocitrate, α-ketoglutarate, and glycerol-3-phosphate. (A) LDH expression begins slightly earlier than other genes associated with the EmbMT. (B) Although levels of lactate and pyruvate appear to increase during embryogenesis, this is diet-dependent (*), and these levels remain largely unchanged when the maternal diet is mostly yeast-based. A pulse (A) of β-oxidation during mid embryogenesis corresponds with a decrease in (B) TAG levels.

We previously demonstrated that the onset of aerobic glycolysis occurs during embryogenesis, when dERR coordinately upregulates nearly every gene that encodes an enzyme in glycolysis as well as *Ldh* (Tennessen *et al.* 2011). This coordinate induction of aerobic glycolysis suggests that *Drosophila* larvae utilize a metabolic program that is similar to the classic Warburg effect model, in which cancer cells exhibit elevated glycolytic flux and decreased oxidative phosphorylation. Here we determine that this transcriptional program is part of a larger metabolic transition, which includes the upregulation of genes encoding TCA cycle enzymes and components of the ETC (Figure 8). Intriguingly, this increase in mitochondrial metabolism is consistent with recent findings in the cancer metabolism field. Although cultured cancer cells are heavily reliant on glucose for biomass production, GBM tumor metabolism *in vivo* deviates from this simple model. Stable isotope tracer analysis, which provides a means to assess the breakdown of glucose within GBM tumors, reveals that glycolysis is coupled to mitochondrial metabolism and oxidative phosphorylation (Maher *et al.* 2012; Marin-Valencia *et al.* 2012). Furthermore, mRNAs that encode ETC components are among the most abundant transcripts present in GBM tumors (Bertagnolli *et al.* 2013), and depletion of ETC-related transcripts inhibits the growth of cancer stem cells (Janiszewska *et al.* 2012).

These observations challenge the more simplistic Warburg effect models and suggest that the coordination of glycolysis and mitochondrial pathways is important for tumor growth. Consistent with this model, even though *Drosophila* larvae exhibit the hallmark metabolic characteristics associated with aerobic glycolysis, mitochondrial metabolism is essential for rapid growth, as many biosynthetic reactions utilize metabolites that are generated by mitochondrial enzymes. Mutations in the citrate synthetase homolog *knockdown* (*kdn*), which generates citrate not only for the TCA cycle but also for *de novo* fatty acid synthesis, slow developmental growth (Fergestad *et al.* 2006). Similarly, decreased ETC activity correlates with slow larval growth and developmental delays (Meiklejohn *et al.* 2013). In contrast, mutations in mitochondrial *malate dehydrogenase* (*Mdh*) have no obvious affect on developmental growth, suggesting that a complete TCA cycle is not required for larval development and illustrating our relatively poor understanding of the relationship between growth and mitochondrial metabolism (Wang *et al.* 2010). These findings suggest that studies of mitochondrial metabolism in embryos may provide new insights into the coordination of glycolysis, mitochondrial pathways, and oxidative metabolism during periods of rapid growth.

### Oxidative stress and metabolic flux

The coordinate changes in metabolic gene expression that we have identified do not necessarily lead to similar changes in metabolic flux (Figure 8). Our metabolomic analysis of embryogenesis revealed the accumulation of several metabolite pools that would not be predicted by the modENCODE data. For example, the levels of citrate, isocitrate, and α-ketoglutarate increase significantly during embryogenesis, whereas succinate exhibits only a modest increase and both fumarate and malate remain largely unchanged. These observations suggest that the TCA cycle is attenuated during embryonic development, possibly to reduce oxidative stress. In addition, gas exchange in embryos is limited to passive diffusion, and embryonic metabolism must ensure that development is not limited by oxygen availability. This model would also explain why embryos exhibit such a dramatic buildup of glycerol-3-phosphate (G3P). The embryonic breakdown of TAG releases three fatty acid molecules and a single glycerol molecule. Glycerol is phosphorylated by glycerol kinase to form G3P, which can then be converted into the glycolytic intermediate dihydroxyacetone phosphate (DHAP) and used by glycolysis. The accumulation of G3P suggests that this latter reaction is unfavorable, and is consistent with the need to reduce a molecule of NAD^+^ to NADH (cytosol) or FAD to FADH_2_ (mitochondria) to drive DHAP production, both of which would be unfavorable reactions in a closed system with limited oxygen availability.

When embryonic metabolism is reexamined in this context, an important parallel emerges between the metabolic transitions that occur in embryos and premalignant cells. Our metabolomic profiles suggest that embryos are adapted to oxidative stress and also that later-stage embryos can respond quickly to oxygen deprivation and can survive in a hypoxia-induced arrested state for more than 8 d (Wingrove and O’Farrell 1999; DiGregorio *et al.* 2001). In contrast, early embryos (stages 1–8) are highly sensitive to hypoxia and die when exposed to short periods of oxygen deprivation (Foe and Alberts 1985), suggesting that a fundamental shift in embryonic metabolism occurs during this time. This transition to hypoxia tolerance is consistent with our metabolomic data. Citrate, isocitrate, α-ketoglutarate, and glycerol-3-phosphate levels are stable for the first 6 hr of embryogenesis but increase rapidly thereafter. Furthermore, the transcriptional upregulation of glycolysis correlates with cuticle synthesis, which acts as an additional barrier for gas exchange. In this context, the increase in glycolytic capacity, providing ATP without oxidative phosphorylation, could favor survival under low oxygen conditions. A similar phenomenon occurs in premalignant cells, where an upregulation of glycolysis coincides with adaptation to oxidative stress (Suchorolski *et al.* 2013).

### Uric acid production is associated with oxidative stress

The finding that uric acid accumulates to high levels in late-stage embryos supports a model in which embryonic metabolism is adapted to oxidative stress. Uric acid is an end product of purine nucleotide degradation and is normally produced when there is either an abundance of free nucleotides or a disruption of other central metabolic pathways. Elevated uric acid production in humans is commonly associated with disorders in which oxidative metabolism is disrupted, such as cardiac failure, acute stroke, sleep apnea, and the onset of type 2 diabetes (Fessel 1980; Leyva *et al.* 1998; Fang and Alderman 2000; Lavie 2003; Weir *et al.* 2003; Bhole *et al.* 2010).

Uric acid production in *Drosophila*, however, not only results from high levels of purine catabolism but also reflects the disposal of nitrogenous waste from amino acid–derived energy production. In this context, the significant depletion of aspartate during embryogenesis is likely linked to the increase in uric acid. Aspartate is one of the three amino acids required for synthesizing purines, and the maternal loading of this amino acid could provide abundant precursor material for nucleotide synthesis. Although the production of ATP during the conversion of amino acid–derived purines into uric acid is rather inefficient, it provides distinct advantages to the developing embryo. Uric acid is highly insoluble and readily forms crystals, which have been observed previously in the developing Malpighian tubules (Skaer 1993). Because embryos are isolated from the external environment, uric acid crystal formation allows for the internal disposal of nitrogenous waste. Furthermore, uric acid is a powerful antioxidant and could protect developing embryos from oxidative stress. Mutants for the *Drosophila* xanthine dehydrogenase homolog *rosy*, which eliminate uric acid production, are sensitive to oxidative stress (Hilliker *et al.* 1992).

Interestingly, uric acid synthesis not only is linked with defects in oxidative metabolism but also is associated with tumor metabolism. Tumor lysis syndrome (TLS) is a life-threatening and unpredictable event that results from tumors producing dangerously high levels of uric acid (Davidson *et al.* 2004). Although the cause remains unclear, TLS often occurs after the administration of chemotherapy, suggesting that uric acid production is a result of metabolic stress. *Drosophila* embryogenesis, therefore, not only provides a unique opportunity to explore the metabolic state that drives uric acid synthesis but also may also provide valuable insights into why stressed tumors produce high levels of uric acid and how this dangerous syndrome might be avoided.

### The role of the EmbMT in embryonic development

The metabolic transitions described here are not used to directly promote larval growth, but rather reflect the metabolic needs of embryonic development. The transcriptional upregulation of these metabolic pathways are likely generating the energy required to drive morphogenesis and synthesizing the biomolecules required to complete embryogenesis. For example, the onset of the EmbMT correlates with the synthesis of the embryonic cuticle, which is largely composed of the molecule chitin and synthesized from fructose-6-phosphate, glutamine, and acetyl-CoA. Many of the enzymes required for chitin synthesis are temporarily upregulated during mid embryogenesis, just prior to the onset of the EmbMT, which together with the upregulation of glycolysis and the TCA cycle could establish an ideal metabolic program for cuticle synthesis. The increased glycolysis during embryonic cuticle synthesis would have the secondary benefit of establishing the metabolic foundation of larval development, allowing newly hatched animals to quickly convert dietary carbohydrates into biomass.

Intriguingly, the EmbMT also occurs during a period of apparent fatty acid metabolism, when embryos are using TAG stores and expressing genes that encode key enzymes involved in TAG breakdown and fatty acid β-oxidation, including the rate-limiting enzyme CPTI (Figure 8). This upregulation of fat metabolism is critical during embryogenesis, as antimorphic mutations in the β-oxidation gene *L-3-hydroxyacyl-CoA dehydrogenase* (*scully*) are embryonic lethal (Torroja *et al.* 1998). The critical role of β-oxidation in embryogenesis, however, does not extend into larval development, as the expression of *CPTI* and other genes involved in β-oxidation are downregulated prior to hatching. Furthermore, mutations in *scully* and *CPTI* do not disrupt larval growth under normal dietary conditions (Torroja *et al.* 1998; Strub *et al.* 2008), suggesting that the completion of embryonic development results in a metabolic switch from fat to carbohydrate metabolism. Intriguingly, this metabolic program is similar to the downregulation of *CPTI* in transformed cancer cells and, again, demonstrates how *Drosophila* development provides a model for studying the metabolic changes that occur as normal cells adopt the abnormal growth program associated with tumorigenesis (Buzzai *et al.* 2005; Deberardinis *et al.* 2006).

## Supplementary Material

Supporting Information
