# Supplementary material for: Coordinated Metabolic Transitions During Drosophila Embryogenesis and the Onset of Aerobic Glycolysis
Source: G3 (Bethesda). 2014 Mar 12;4(5):839–50. doi: 10.1534/g3.114.010652 (PMC4025483; doi:10.1534/g3.114.010652)
Supplement: Supporting Information [file supp_4_5_839__index.html]

Coordinated Metabolic Transitions During Drosophila Embryogenesis and the Onset of Aerobic Glycolysis — Supporting Information 

# Coordinated Metabolic Transitions During *Drosophila* Embryogenesis and the Onset of Aerobic Glycolysis

## Supporting Information for Tennessen *et al.*, 2014

**Files in this Data Supplement:**

- Supporting Information - Figures S1-S3, File S1, and Tables S1-S8 (PDF, 194 KB)
- Figure S1 - Metabolic genes present in SVD patterns. (PDF, 121 KB)
- Figure S2 - Stored lipids and carbohydrates are depleted during *w1118* embryogenesis. (PDF, 119 KB)
- Figure S3 - Maternal diet affects glycolysis in *Canton-S* embryos. (PDF, 125 KB)
- File S1 - Supplemental Methods (.zip, 425 KB)
- Table S1 - SVD analysis of the ModENCODE embryonic RNAseq timecourse. (.xlsx, 3 MB)
- Table S2 - List of *Drosophila* metabolic genes. (.xlsx, 99 KB)
- Table S3 - Metabolic genes represented by SVD pattern 2. (.xlsx, 37 KB)
- Table S4 - GO categories correlated with SVD pattern 3. (.xlsx, 53 KB)
- Table S5 - Metabolic genes correlated with SVD pattern 3. (.xlsx, 33 KB)
- Table S6 - Metabolomic analysis of *w1118* embryogenesis, yeast and molasses agar as food source (raw values). (.xlsx, 577 KB)
- Table S7 - Metabolomic analysis of *Canton-S* embryogenesis, Experiment 1, yeast and molasses agar as food source (raw values). (.xlsx, 93 KB)
- Table S8 - Metabolomic analysis of *Canton-S* embryogenesis, Experiment 2, semi-defined media as a food source (raw values). (.xlsx, 147 KB)
