# Supplementary material for: Coordinated Metabolic Transitions During Drosophila Embryogenesis and the Onset of Aerobic Glycolysis
Source: G3 (Bethesda). 2014 Mar 12;4(5):839–50. doi: 10.1534/g3.114.010652 (PMC4025483; doi:10.1534/g3.114.010652)
Supplement: Supporting Information [file supp_g3.114.010652_010652SI.pdf]

G3 · Genes | Genomes | Genetics

Coordinated metabolic transitions during *Drosophila* embryogenesis and the onset of aerobic glycolysis

Jason M. Tennessen[1,2*], Nicolas M. Bertagnolli[3,4], Janelle Evans[1], Matt H. Sieber[1,5], James Cox[6], and Carl S. Thummel[1]

[1] Department of Human Genetics, University of Utah School of Medicine, 15 N 2030 E Rm 2100, Salt Lake City, UT 84112 USA
[2] Present address: Department of Biology, Indiana University, 1001 East Third Street, Bloomington, IN 47405
[3] Scientific Computing and Imaging (SCI) Institute, University of Utah, Salt Lake City, UT 84112
[4] Department of Mathematics, University of Utah, Salt Lake City, UT 84112, USA
[5] Present address: Department of Embryology, Carnegie Institution for Science, Baltimore, MD 21218
[6] Department of Biochemistry and the Metabolomics Core Research Facility, University of Utah School of Medicine, Salt Lake City, UT 84112, USA

*Correspondence: jtenness@indiana.edu

DOI: 10.1534/g3.114.010652

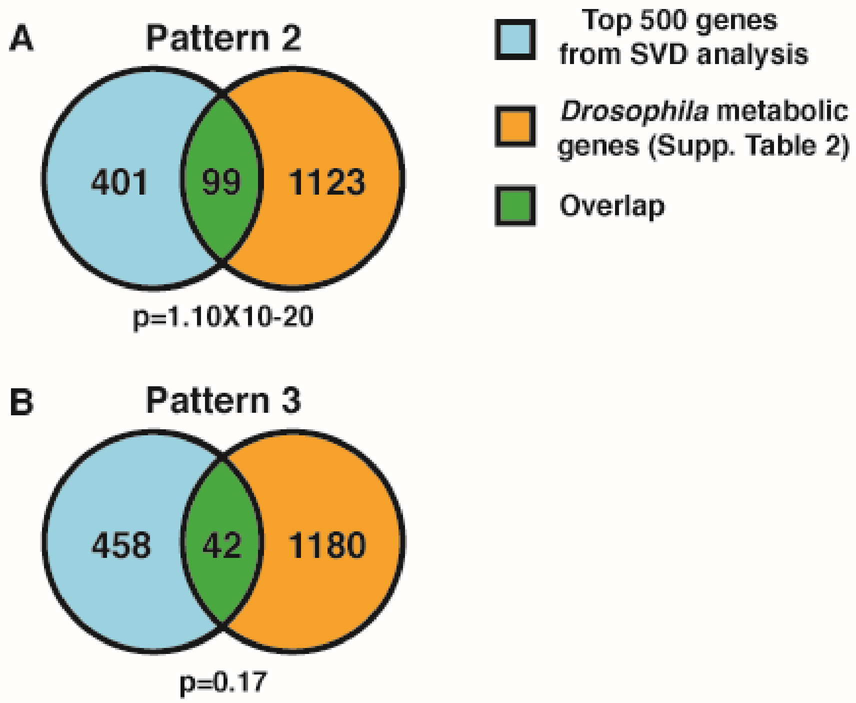

Figure S1 Metabolic genes present in SVD patterns. The top 500 genes associated with SVD patterns 2 and 3 were analyzed for the presence of known metabolic genes (Supplemental Table 2). (A) Pattern 2 exhibits a significant enrichment of metabolic genes, but (B) Pattern 3 does not. p value calculated using hypergeometric distribution.

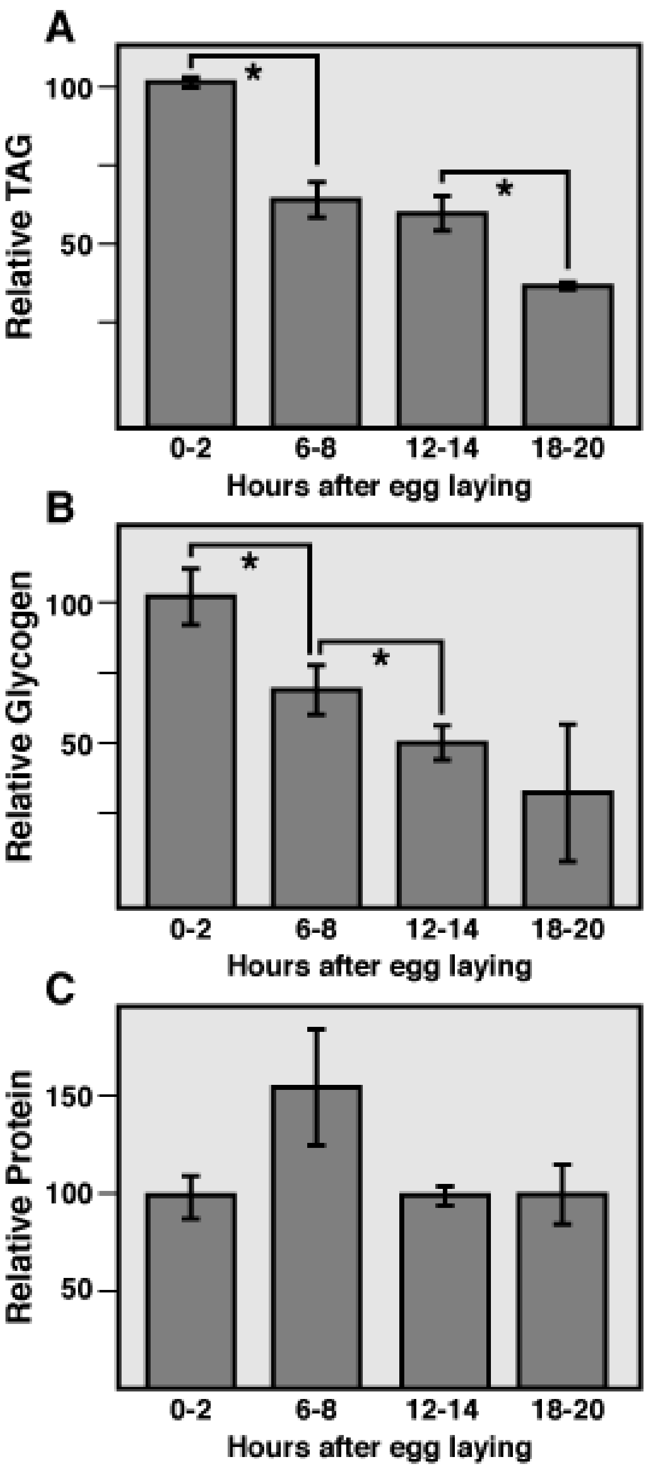

Figure S2 Stored lipids and carbohydrates are depleted during w^{1118} embryogenesis. (A) Triacylglycerol (TAG), (B) glycogen, and (C) soluble protein levels were measured at four-hour intervals during the course of embryogenesis. Both TAG and glycogen levels significantly decrease as development progresses, while protein levels moderately increase during this time (* indicates that $p<0.002$, Student's T-test). Each bar represents n=6 samples containing 300 staged and hand-sorted w^{1118} embryos. Error bars represent ±SEM.

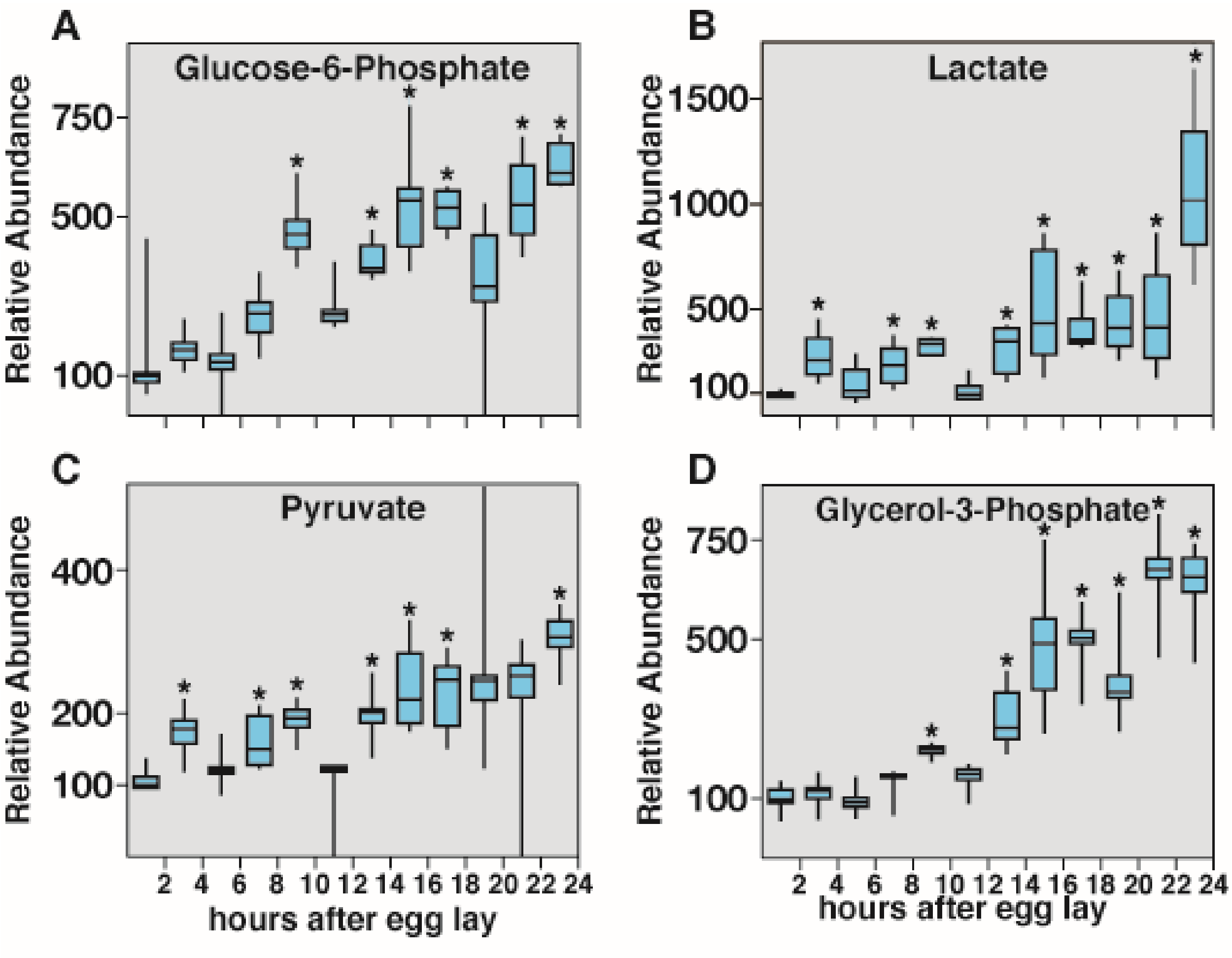

Figure S3 Maternal diet affects glycolysis in *Canton-S* embryos. Small molecule GC/MS was used to analyze the relative abundance of metabolites in embryos from mothers raised on semi-defined medium. The increased sugar concentration in the maternal diet correlates with increasing levels of (A) glucose-6-phosphate and (B) lactate during embryogenesis. (C) Pyruvate levels exhibited a modest increase during the time course. Meanwhile, similar to the w^{1118} experiments, (D) glycerol-3-phosphate levels increase at a constant rate. * indicates that p<0.01 compared with 0-2 hr timepoint; Student's T-test. All data are graphically represented as described in legend for Figure 4.

File S1

Supplemental Methods

File S1 is available for download as a .zip file at http://www.g3journal.org/lookup/suppl/doi:10.1534/g3.114.010652/-/DC1

Tables S1-S8

Available for download as Excel files at http://www.g3journal.org/lookup/suppl/doi:10.1534/g3.114.010652/-/DC1

Table S1 SVD analysis of the ModENCODE embryonic RNAseq timecourse

Table S2 List of *Drosophila* metabolic genes

Table S3 Metabolic genes represented by SVD pattern 2

Table S4 GO categories correlated with SVD pattern 3

Table S5 Metabolic genes correlated with SVD pattern 3

Table S6 Metabolomic analysis of w^{1118} embryogenesis, yeast and molasses agar as food source (raw values)

Table S7 Metabolomic analysis of *Canton-S* embryogenesis, Experiment 1, yeast and molasses agar as food source (raw values)

Table S8 Metabolomic analysis of *Canton-S* embryogenesis, Experiment 2, semi-defined media as a food source (raw values)
