# Supplementary material for: Coordinated Metabolic Transitions During Drosophila Embryogenesis and the Onset of Aerobic Glycolysis
Source: G3 (Bethesda). 2014 Mar 12;4(5):839–50. doi: 10.1534/g3.114.010652 (PMC4025483; doi:10.1534/g3.114.010652)
Supplement: Supporting Information [file supp_g3.114.010652_FigureS1.pdf]

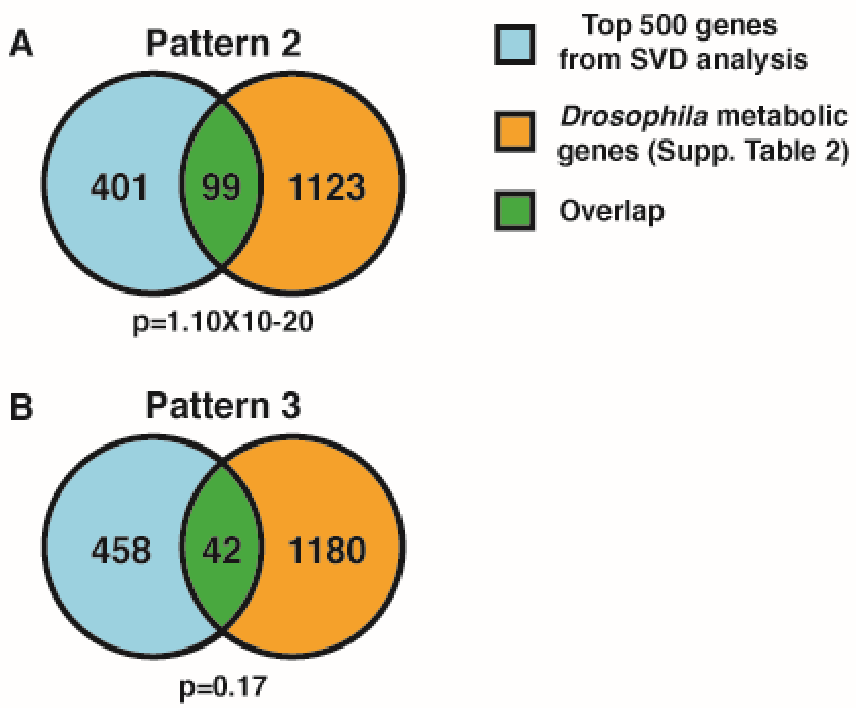

Figure S1 Metabolic genes present in SVD patterns. The top 500 genes associated with SVD patterns 2 and 3 were analyzed for the presence of known metabolic genes (Supplemental Table 2). (A) Pattern 2 exhibits a significant enrichment of metabolic genes, but (B) Pattern 3 does not. p value calculated using hypergeometric distribution.
