# Supplementary material for: Coordinated Metabolic Transitions During Drosophila Embryogenesis and the Onset of Aerobic Glycolysis
Source: G3 (Bethesda). 2014 Mar 12;4(5):839–50. doi: 10.1534/g3.114.010652 (PMC4025483; doi:10.1534/g3.114.010652)
Supplement: Supporting Information [file supp_g3.114.010652_FigureS2.pdf]

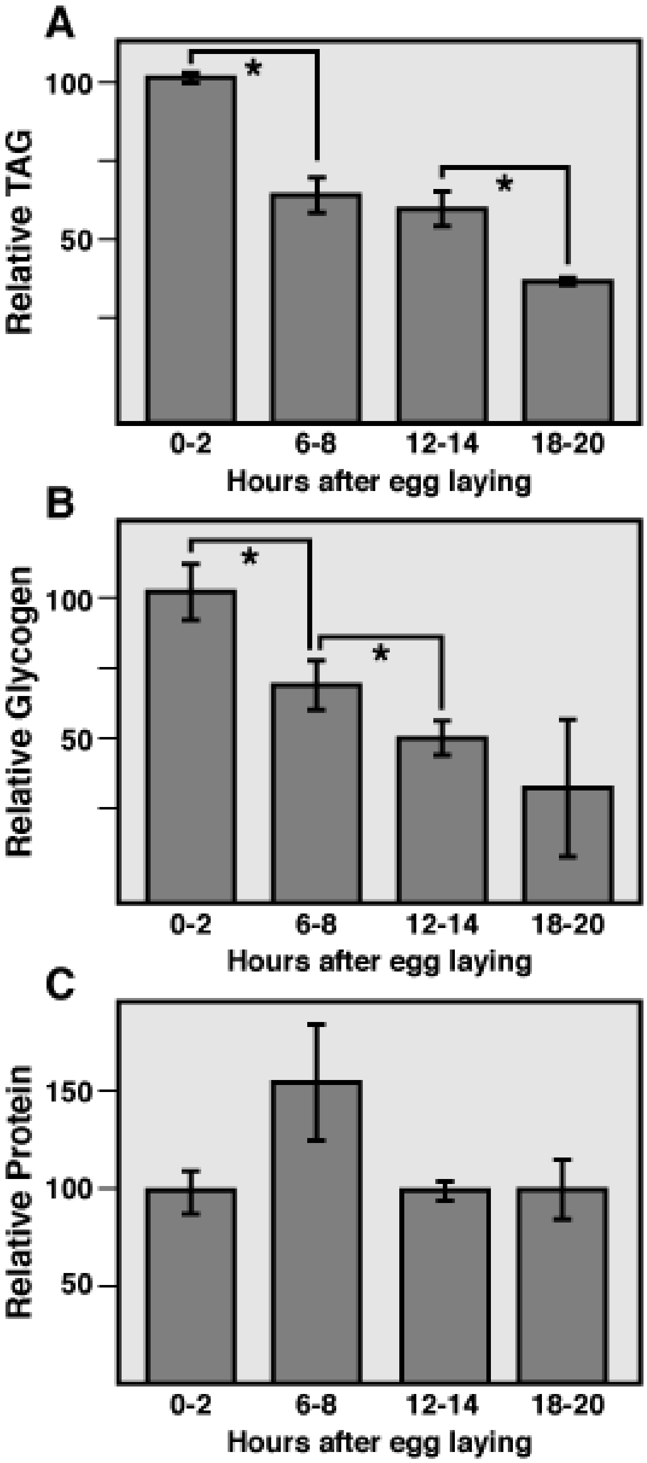

Figure S2 Stored lipids and carbohydrates are depleted during *w*[1118] embryogenesis. (A) Triacylglycerol (TAG), (B) glycogen, and (C) soluble protein levels were measured at four-hour intervals during the course of embryogenesis. Both TAG and glycogen levels significantly decrease as development progresses, while protein levels moderately increase during this time (* indicates that $p<0.002$, Student's T-test). Each bar represents n=6 samples containing 300 staged and hand-sorted *w*[1118] embryos. Error bars represent ±SEM.
