# Supplementary material for: Coordinated Metabolic Transitions During Drosophila Embryogenesis and the Onset of Aerobic Glycolysis
Source: G3 (Bethesda). 2014 Mar 12;4(5):839–50. doi: 10.1534/g3.114.010652 (PMC4025483; doi:10.1534/g3.114.010652)
Supplement: Supporting Information [file supp_g3.114.010652_FigureS3.pdf]

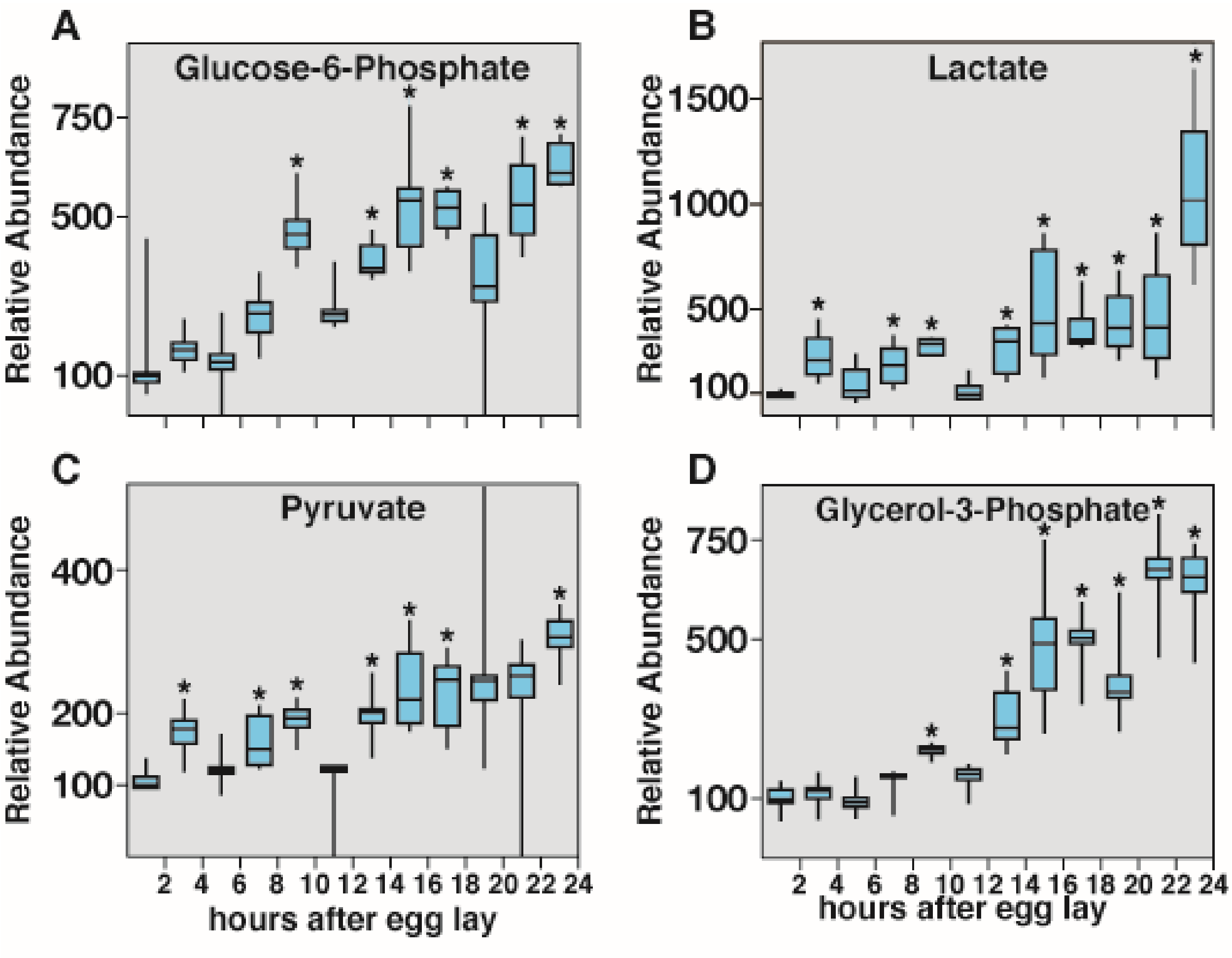

Figure S3 Maternal diet affects glycolysis in *Canton-S* embryos. Small molecule GC/MS was used to analyze the relative abundance of metabolites in embryos from mothers raised on semi-defined medium. The increased sugar concentration in the maternal diet correlates with increasing levels of (A) glucose-6-phosphate and (B) lactate during embryogenesis. (C) Pyruvate levels exhibited a modest increase during the time course. Meanwhile, similar to the w^{1118} experiments, (D) glycerol-3-phosphate levels increase at a constant rate. * indicates that p<0.01 compared with 0-2 hr timepoint; Student's T-test. All data are graphically represented as described in legend for Figure 4.
