# Supplementary figures and images for: Coordinated Metabolic Transitions During Drosophila Embryogenesis and the Onset of Aerobic Glycolysis
Source: G3 (Bethesda). 2014 Mar 12;4(5):839–50. doi: 10.1534/g3.114.010652 (PMC4025483; doi:10.1534/g3.114.010652)

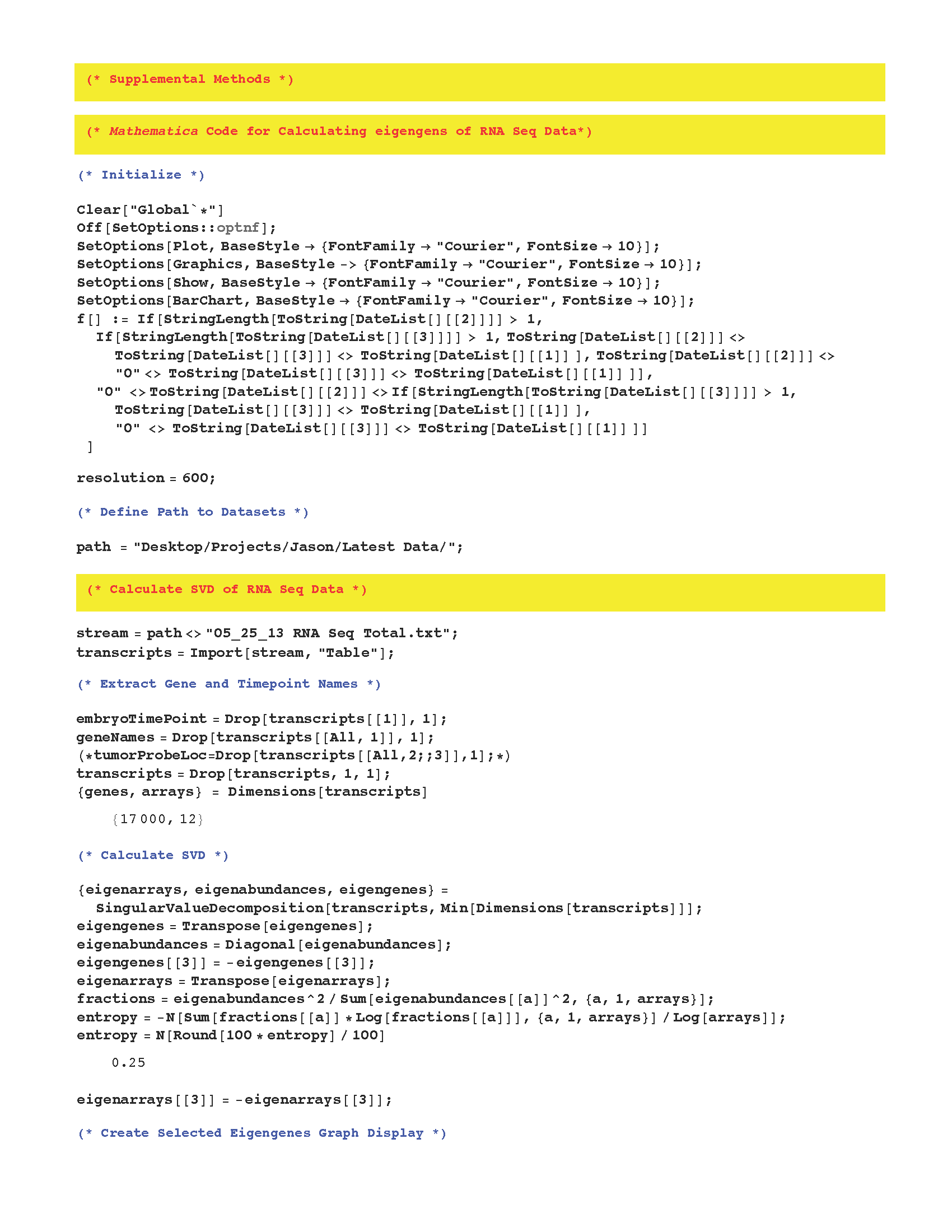

Supplement: Supporting Information [file supp_g3.114.010652_FileS1.zip › FileS1/Supplemental Methods_Page_1.tiff]

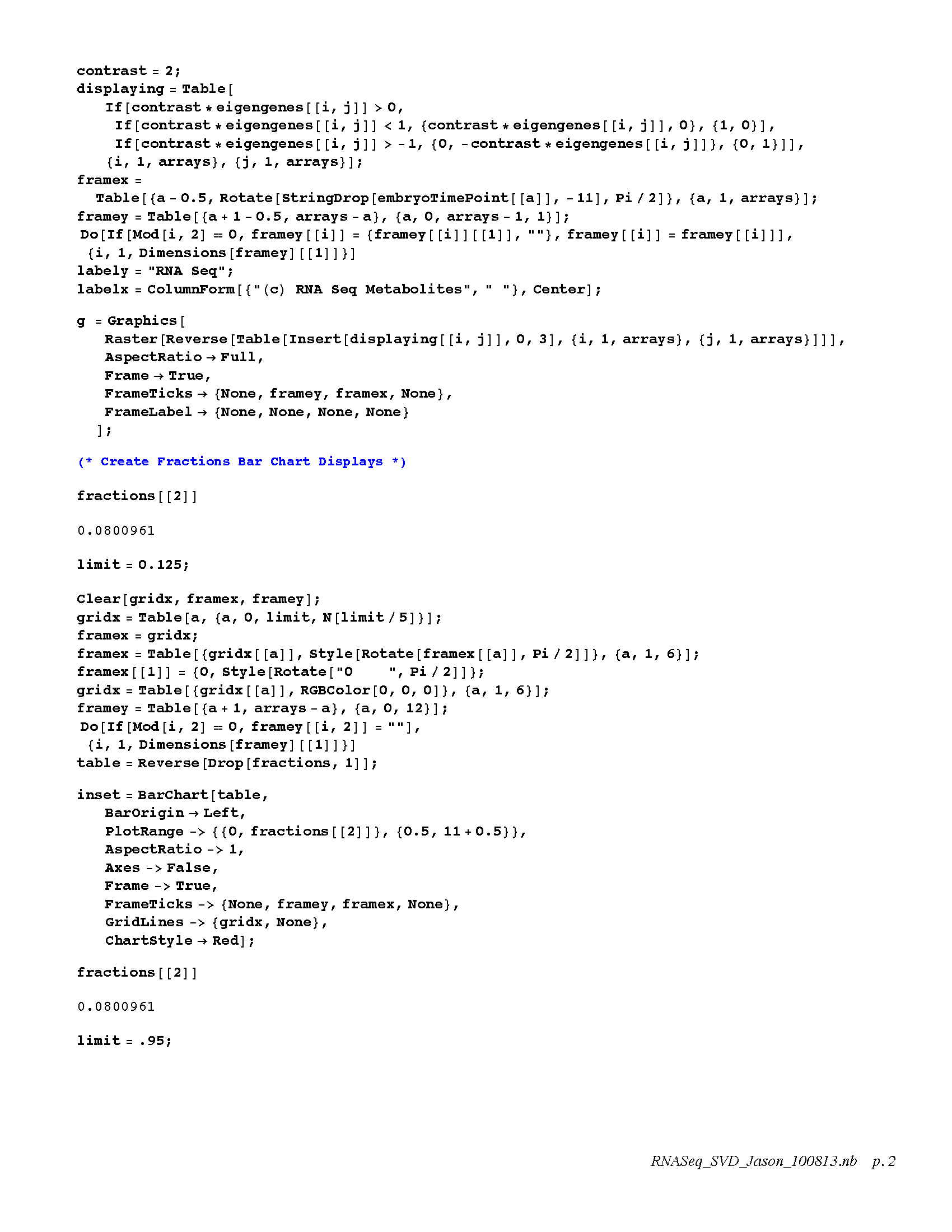

Supplement: Supporting Information [file supp_g3.114.010652_FileS1.zip › FileS1/Supplemental Methods_Page_2.tiff]

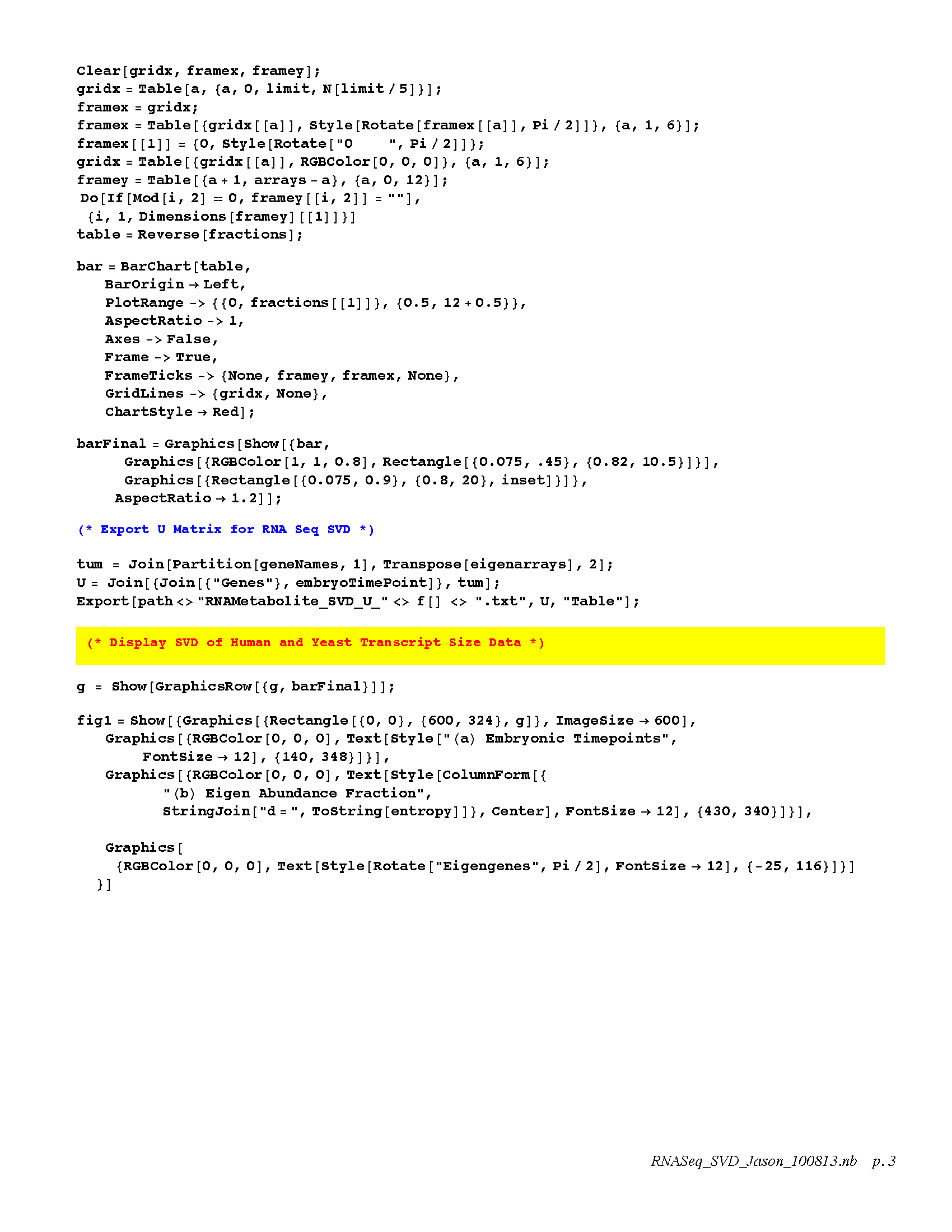

Supplement: Supporting Information [file supp_g3.114.010652_FileS1.zip › FileS1/Supplemental Methods_Page_3.tiff]

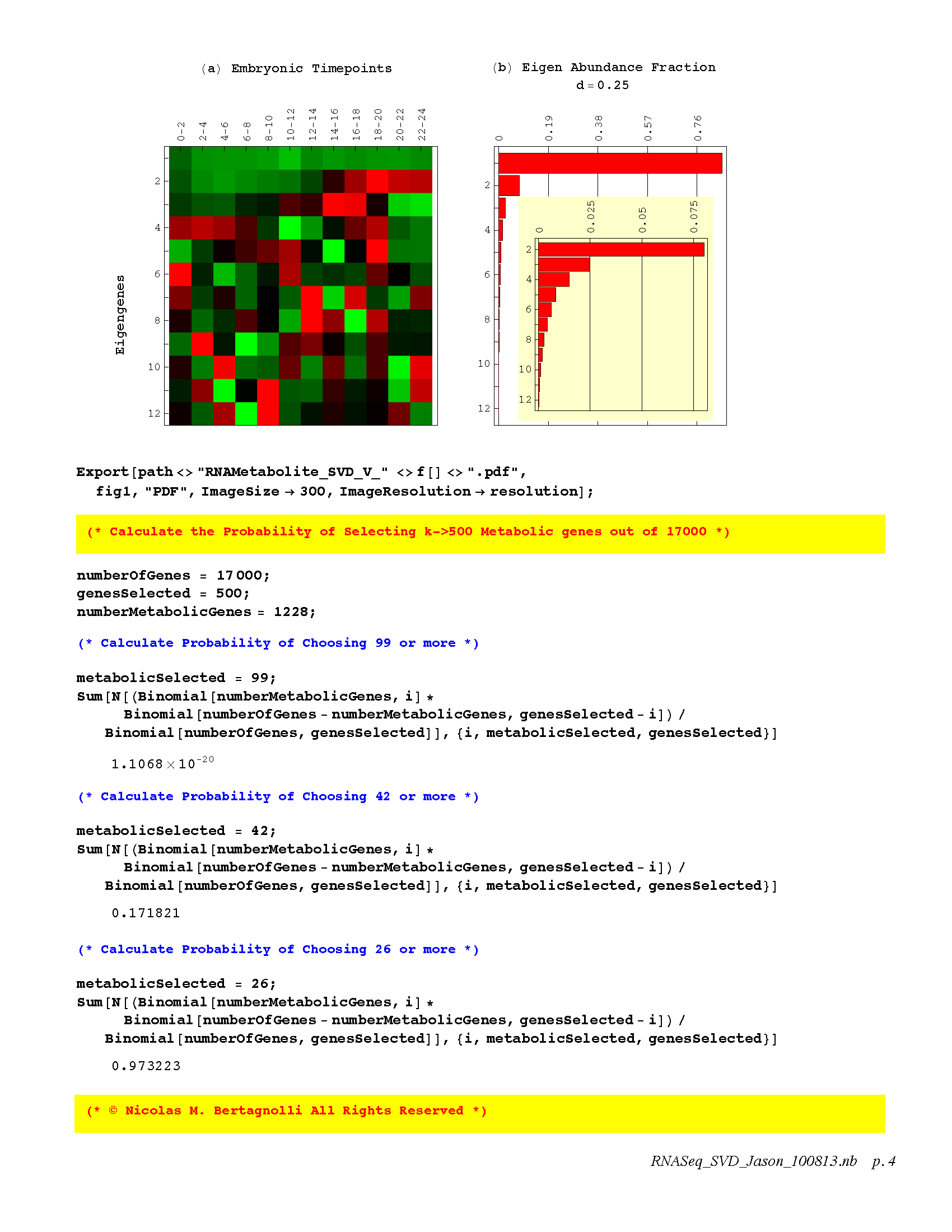

Supplement: Supporting Information [file supp_g3.114.010652_FileS1.zip › FileS1/Supplemental Methods_Page_4.tiff]
